# Association of the Thrombo-embolic Phenomenon with Electroconvulsive Therapy Treatment in Schizophrenia with Catatonia Patient

**DOI:** 10.7759/cureus.5656

**Published:** 2019-09-14

**Authors:** Zain I Warriach, Sohaib A Shamim, Aisha Saeed, Saima Kashif, Bilal Haider Malik

**Affiliations:** 1 Psychiatry, California Institute of Behavioral Neurosciences and Psychology, Fairfield, USA; 2 Neurology, California Institute of Behavioral Neurosciences and Psychology, Fairfield, USA; 3 Family Medicine, California Institute of Behavioral Neurosciences and Psychology, Fairfield, USA; 4 Department of Research, California Institute of Behavioral Neurosciences and Psychology, Fairfield, USA; 5 Internal Medicine, California Institute of Behavioral Neurosciences and Psychology, Fairfield, USA

**Keywords:** ect, thrombosis, catatonia, schizophrenia

## Abstract

Catatonia is a condition in which patients manifest with a complex of symptoms of behavioral and motor abnormalities. This condition can present with schizophrenia, bipolar, depression, and certain neurological illnesses. In this article, we analyze the coincidence of deep venous thrombosis (DVT) in schizophrenia with catatonia patients managed with electroconvulsive therapy (ECT) and the possible outcome of complications as an adverse event. The conclusion drawn from this traditional review reveals the importance of early diagnosis and treatment intervention of catatonia patients with ECT. No bleeding-related complications occurred with the use of anticoagulants by catatonic patients managed with ECT and having underlying thrombosis. Literature also indicates that ECT can be safely administered for patients with distal deep venous thrombosis (DVT) treated with anticoagulants, while those with proximal DVT, ECT should be halted and anticoagulation treatment should be continued until the resolution of DVT to safely resume the ECT sessions. A possible alternative to ECT therapy for the patients with refractory schizophrenia with catatonia (who have safety concerns) is recombinant transcranial magnetic stimulation (rTMS). Nonetheless, more studies are needed to support our assertion.

## Introduction and background

This traditional review deals with the importance of knowing the association between thrombosis and long-term schizophrenia with catatonia patients and the further association of thrombo-embolic phenomenon with electroconvulsive therapy (ECT) in schizophrenia with catatonia patients.

Catatonia is a state of motor and behavioral abnormality caused by chronic psychiatric conditions especially schizophrenia. It is a very debilitating condition manifested by various symptoms of stupor, rigidity, posturing, mutism, negativism and waxy flexibility which requires early intervention and treatment to avoid resistance to treatment [1, 2]. The main treatment protocol for catatonia is comprised of benzodiazepines and for severe unresponsive cases, ECT is recommended.

One of the established facts regarding chronic psychiatric conditions is the outcome of thrombosis due to immobility, dehydration, malnutrition, venous stasis and iatrogenesis like physical restraints, tranquilizers, and antipsychotics medication usage [3-5]. Various other complications can arise in catatonic patients if left untreated for longer periods like pressure ulcers, aspiration pneumonia, urinary retention and infection which should be addressed immediately to prevent long term consequences in these patients. Therefore, early treatment intervention of catatonia with the use of benzodiazepines and possible ECT is important to prevent these complications and possible treatment resistance [6, 7].

ECT is the definitive treatment for refractory cases of schizophrenia with catatonia having underlying thrombosis [8]. The severe muscle contraction during ECT session can dislodge the clot from residual deep venous thrombosis and embolize to other organs increasing the risk of pulmonary embolism. Literature shows that schizophrenia with catatonic patients having underlying proximal deep venous thrombosis (DVT) and managed with ECT develop pulmonary embolism as a complication while those catatonic patients with distal deep venous thrombosis and managed with ECT do not develop any complication [9].

This review article is very important and informative for the attending psychiatrist in following the treatment strategy of schizophrenia with catatonic patients, particularly with underlying deep venous thrombosis. It also shows the importance of early diagnosis and treatment of catatonia with ECT and safe continuation of the anticoagulation treatment along with ECT. It further serves as a guide to evaluating the benefits and risks of conducting ECT in patients with schizophrenia with catatonia with underlying DVT and finding alternative treatment strategy and to conclude the overall benefits of avoiding treatment resistance and debilitation in a catatonic patient versus further deterioration and consequences by increasing the chances of embolization.

## Review

This is a traditional review, not a systematic review, so no Preferred Reporting Items for Systematic Reviews and Meta-Analyses (PRISMA) or Measurement Tool to Assess Systematic Reviews (AMSTAR) guidelines are used. Reference databases are used mostly from PubMed and a few from Google scholar. No inclusion and exclusion criteria are used for the review article. Regular key-words - “thrombosis” and “ECT” - yield 40 articles on PubMed. While regular keywords - “catatonia” and “thrombosis” and “ECT” - showed four articles. The Medical Subject Headings (MeSH) keywords for these searches yield no articles. As healthcare professionals, we collected and used all the data ethically.

This traditional review deals with the efficacy, safety and potential adverse events associated with the management of chronic schizophrenia with catatonia patients with electroconvulsive therapy (ECT) and underlying thrombosis. Catatonia is a syndrome which is manifested by various behavioral and motor abnormalities, usually episodic in nature with remissions [1]. The patients affected with catatonia often cannot provide a thorough history and therefore the history should be obtained from personal and professional collaterals as well as observing the behavioral and motor abnormalities. Undiagnosed catatonia poses a serious morbidity and mortality risk and should be diagnosed early to prevent treatment resistance and mortality from various complications of catatonia ranging from neurological, psychiatric and medical-related. The American Psychiatric Association’s Diagnostic and Statistical Manual of Mental Disorders, Fifth Edition (DSM-5) categorizes catatonia into three different categories:

1. catatonia associated with another mental disorder (catatonia specifier);

2. catatonia disorder due to another medical condition;

3. unspecified catatonia.

DSM-5 diagnostic criteria for catatonia involves the presence of three of the following twelve features of behavioral and motor abnormality: stupor, catalepsy, waxy flexibility, mutism, negativism, posturing, mannerism, stereotyping, agitation, grimacing, echolalia, echopraxia along with other guidelines of DSM-5.

Pathophysiology and etiology of catatonia

Catatonia usually involves comorbid psychiatric disorders like schizophrenia, bipolar and depressive disorder, dysfunctions of neurotransmitters like dopamine, gamma-aminobutyric acid (GABA) and others in brain circuits, genetics, and inherited influences. There are also numerous medical, neurological, and obstetrical conditions associated with catatonia presentation. The Bush Francis Catatonia Rating Scale (BFCRS) should be used to assess the severity of catatonia along with other laboratory tests, imaging studies and electroencephalogram (EEG) to rule out other conditions.

Pathophysiology of thrombosis in chronic psychiatric conditions like catatonia

One of the most morbid complications of catatonia is thrombosis. It may arise due to immobility, venous stasis, malnutrition and iatrogenesis factors like physical restraints, use of sedatives and antipsychotic medications [3]. Chronic psychiatric patients usually have an unhealthy lifestyle due to economic and social factors. The lack of exercise and side effects of psychiatric medications contribute to obesity, hyperlipidemia, and diabetes, which directly increases their risk of thrombosis [4]. Fibrin D-dimer levels are usually increased in catatonic patients and must be routinely checked to rule out early activation of coagulation in the patient. Any susceptible patient should be ruled out via Doppler ultrasound and anticoagulation treatment should be started at the earliest to resolve deep venous thrombosis [5].

Treatment of schizophrenia with catatonia

Pharmacotherapy like benzodiazepines, carbamazepine, tricyclic antidepressants, and atypical antipsychotics are used for the treatment of catatonia [6]. For refractory cases not responding to medications, electroconvulsive therapy is implemented.

The pros and cons of ECT treatment in schizophrenia with catatonia

In schizophrenia with catatonia resistant to medical treatment, ECT is recommended as a definitive treatment. Only a few cases of catatonic patients responded to pharmacological treatment completely while the majority of patients responded and returned to remission state with ECT treatment. The benefit of early ECT is to prevent life-threatening complications associated with the catatonia like trauma, malnutrition, autonomic instability, pressure ulcers, urinary retention, neuroleptic syndrome (NMS) and complications from the underlying medical, neurological, obstetrical, psychological and psychiatric causes of catatonia [7]. There are, however, some adverse events associated with ECT including confusion, memory loss, physical symptoms like headache, jaw pain or muscle ache, medical complications in underlying heart disease and thromboembolism. 

Pathophysiology of thromboembolism with ECT treatment

**Figure 1:**
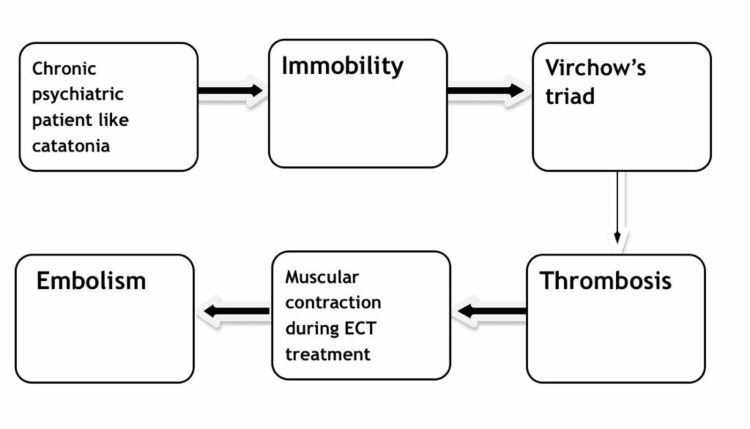
Flowsheet describing the occurrence of thromboembolism with electroconvulsive therapy (ECT) treatment in schizophrenia with catatonia patient

Patients with Virchow’s triad risk factors like hypercoagulability, stasis and endothelial injury are at risk for venous thrombosis. In chronically ill psychiatric patients like schizophrenia with catatonia who remain bedridden for long periods, there is increased propensity for thrombosis [8]. During the ECT session, strong muscular contractions take place which can increase the chances of embolism to other organs usually the lungs leading to ventilation/perfusion mismatch, increased physiological dead space, hypocapnia, and atelectasis resulting in dyspnea and tachypnea. If untreated, large pulmonary embolism can cause death by the increase in the right ventricular pressure and ultimate right heart failure. Figure 1 below describes the occurrence of thromboembolism with ECT treatment in schizophrenia with catatonia patient.

There has been literature published looking at psychiatric patients diagnosed with catatonia and underlying thrombosis. There have been incidences described in the literature where catatonic patients developed deep venous thrombosis and anticoagulation treatment was given to all of them before and during ECT sessions and, consequently, no bleeding-related complications were noted. Literature also suggests that the location of deep venous thrombosis might prove to be an important consideration in these patients as catatonic patients with proximal deep venous thrombosis are more likely to develop pulmonary embolism as a complication as compared to catatonic patients with distal deep venous thrombosis [9]. All of these catatonic patients were given muscle relaxants before the ECT session, so it could not be confirmed whether ECT was directly responsible for pulmonary embolism in one of the patients or other additional factors [9-12]. Another literature review highlights three important points that ECT can be administered safely in the psychiatric patient with catatonia after the occurrence of pulmonary embolism, that is, by evaluation of cardiac function and residual deep venous thrombosis before resuming ECT therapy, adjustment of anticoagulation dose and by adopting preventive measures for recurrent deep venous thrombosis and pulmonary embolism like fluid administration, compression devices and timely ECT [13]. The further literature review also supports the safety of ECT therapy in patients receiving anticoagulants for underlying deep venous thrombosis [14-16]. Catatonia with its thromboembolic complication can act as a silent killer and can be easily mistaken for sudden cardiac arrest. It is, therefore, very important to diagnose these lethal complications in a catatonic patient early on in the course of illness [17]. The literature also emphasizes the importance of early diagnosis of catatonia and its treatment with ECT to prevent life-threatening complications. It has also been suggested in the literature that catatonic patients suffering from malnutrition and immobility in addition to deep venous thrombosis can be successfully treated with ECT [18]. During pregnancy and puerperium period, thromboembolism can occur as a possible complication. Our literature search also indicated the successful use of ECT in a female patient with post-partum psychosis with catatonia secondary to cortical venous thrombosis. She had also developed focal neurological deficits as a complication. Due to relapse on the benzodiazepine, she was managed with ECT therapy and responded well to the treatment and her neurological complications also improved [19].

From our review of literature, we derive the following clinical points, treatment strategies, and alternative treatment for the patients with refractory schizophrenia with catatonia and underlying thrombosis:

1. The location of DVT has prime importance before the application of the ECT procedure in schizophrenia with catatonic patients. In patients with distal DVT, ECT sessions can be safely done without any complications with the continuation of anticoagulation, while for patients with proximal DVT, ECT should be stopped and anticoagulation treatment should be continued until the resolution of deep venous thrombosis to safely resume the ECT.

2. Anticoagulation treatment can be safely administered for DVT patients undergoing ECT therapy without bleeding complications. 

3, Routine screening of DVT should be done regularly with D-dimer and Doppler ultrasound in chronic psychiatric patients, especially in patients with a catatonic presentation.

4, Early diagnosis of catatonia and definitive treatment with ECT therapy is very important to prevent life-threatening complications in patients. 

5. Recombinant transcranial magnetic stimulation (rTMS) could be a potential alternative for the treatment of refractory schizophrenia with catatonia when there are safety concerns with ECT [20].

Table 1 below lists important studies which are relevant to the review article

**Table 1:** Important studies which are relevant to the review article ECT - electroconvulsive therapy; DVT - deep venous thrombosis

#	Author	Year of publication	Country of origin of the study	Important points of the study
1	Inagawa Y [9]	2018	Japan	ECT can be safely administered for patients with distal DVT, while in cases of proximal DVT, ECT should be halted and anticoagulation treatment should be continued until the resolution of DVT to safely resume the ECT sessions.
2	Kar SK [19]	2013	India	A case of post-partum psychosis with catatonia as a major clinical presentation. The patient is treated with ECT due to relapse on benzodiazepine.
3	Medda P [18]	2012	Italy	A case of catatonia patient with complications like deep venous thrombosis, pressure ulcers and septic syndrome due to delayed management, treated successfully with ECT.
4	Suzuki K [13]	2008	Japan	Two patients with psychiatric disorders of depression and catatonia which are complicated by pulmonary embolism and treated successfully with ECT.

## Conclusions

The conclusion drawn from this traditional review is the importance of the treatment of refractory schizophrenia with catatonic patients with electroconvulsive therapy. Patients with distal deep venous thrombosis can be safely administered with ECT therapy with the continuation of anticoagulation, while those with proximal DVT, ECT should be halted and anticoagulation treatment should be continued until the resolution of DVT to safely resume the ECT sessions. Early diagnosis and treatment of schizophrenia with catatonia patients with ECT therapy is recommended to prevent life-threatening complications in the patient. Recombinant transcranial magnetic stimulation (rTMS) could be a potential alternative for the treatment of refractory schizophrenia with catatonia when there are safety concerns with ECT therapy. Nonetheless, more studies are needed to support our assertion. 
